# Imaging of Strong Nanoscale Vortex Pinning in GdBaCuO High-Temperature Superconducting Tapes

**DOI:** 10.3390/nano11051082

**Published:** 2021-04-22

**Authors:** David Collomb, Min Zhang, Weijia Yuan, Simon J. Bending

**Affiliations:** 1 Department of Physics, University of Bath, Claverton Down, Bath BA2 7AY, UK; pyssb@bath.ac.uk; 2 Applied Superconductivity Laboratory, Department of Electronics and Electrical Engineering, University of Strathclyde, Glasgow G1 1XQ, UK; min.zhang@strath.ac.uk (M.Z.); weijia.yuan@strath.ac.uk (W.Y.)

**Keywords:** high-temperature superconducting tapes, magnetic imaging, nanoscale defects, cuprates, scanning hall microscopy

## Abstract

The high critical current density of second-generation high-temperature superconducting (2G-HTS) tapes is the result of the systematic optimisation of the pinning landscape for superconducting vortices through careful engineering of the size and density of defects and non-superconducting second phases. Here, we use scanning Hall probe microscopy to conduct a vortex-resolved study of commercial GdBaCuO tapes in low fields for the first time and complement this work with “local” magnetisation and transport measurements. Magnetic imaging reveals highly disordered vortex patterns reflecting the presence of strong pinning from a dense distribution of nanoscale Gd_2_O_3_ second_-_phase inclusions in the superconducting film. However, we find that the measured vortex profiles are unexpectedly broad, with full-width-half-maxima typically of 6 μm, and exhibit almost no temperature dependence in the range 10–85 K. Since the lateral displacements of pinned vortex cores are not expected to exceed the superconducting layer thickness, this suggests that the observed broadening is caused by the disruption of the circulating supercurrents due to the high density of nanoscale pinning sites. Deviations of our local magnetisation data from an accepted 2D Bean critical state model also indicate that critical state profiles relax quite rapidly by flux creep. Our measurements provide important information about the role second-phase defects play in enhancing the critical current in these tapes and demonstrate the power of magnetic imaging as a complementary tool in the optimisation of vortex pinning phenomena in 2G-HTS tapes.

## 1. Introduction

Cuprate superconductors have been the subject of intense investigation ever since their discovery due to their promising application as the superconducting layer in high-temperature superconducting (HTS) coated conductors, the second generation (2G) of which are known as 2G-HTS tapes. These typically have superconducting critical temperatures, *T_c_*, greater than the boiling point of liquid nitrogen (77 K), and high superconducting critical current densities, *J_c_*. Such coated conductors hold great promise, with possible applications in electrical power transmission [1], fault current limiters [2], superconducting motors and generators [3], high-field magnets above 20T [4,5], Maglev-based transport and superconducting magnetic energy storage [6,7]. A major challenge for the adoption of HTS tapes in some of these applications is the need to overcome the rapid deterioration of *J_c_* in high magnetic fields. If a supercurrent is applied in the mixed state of these type-II superconductors, when the superconductor is between the lower critical field, H_c1_, and upper critical field, H_c2_, magnetic flux lines threading the material will move as a result of the Lorentz force. This motion leads to finite energy dissipation, resulting in the loss of the zero resistance state. In practice, these flux lines are pinned at normal “defects” in the superconducting layer including impurities, second phases, grain boundaries and dislocations. Since the Lorentz force scales linearly with the supercurrent density, keeping these flux lines pinned at very high currents requires careful engineering of the pinning sites, controlling their size and density to optimise the energy landscape for the maximum critical current density. Ideally, pinning centres should have sizes of the order of the vortex core diameter; if they are smaller than the superconducting coherence length, *ξ*, the vortex core may spread to other sites, resulting in a reduction in the net pinning forces. There are several ways manufacturers can incorporate artificial pinning centres into cuprate superconductors. These include the addition of normal rare-earth precipitates [8,9], chemical modification through ion irradiation [10], surface deformation [11] and substrate surface decoration [12]. Advances in tape performance must also be combined with a low cost and the ability to produce long lengths with high throughput, as reflected in a price-performance ratio [13]. The task is made particularly challenging due to the complex interplay between flux lines and the many different pinning sites that can be routinely introduced in 2G-HTS tapes. Furthermore, any one type of pinning site may not be equally effective across all temperature and magnetic field regimes. Pinning centres can be assigned to different categories depending on their dimensionality from 0D to 3D [13,14]. Examples of 0D pinning sites are point-like defects whose size is on the order of the coherence length or smaller such as oxygen vacancies, elemental substitutions and point defects. At a larger scale, 1D pinning sites are extended linear defects such as columnar defects or dislocations whose two short dimensions are of the order of the superconducting coherence length. Planar defects such as grain boundaries and twin boundaries constitute 2D pinning sites, while 3D pinning sites are normally large bulk defects such as second phases, voids or precipitates, whose sizes greatly exceed the superconducting coherence length. One example of how these can be tailored for applications is in the case of high-field magnets for operation at liquid helium temperatures. Here, 0D pinning sites are often desirable as their impact is still significant at these low temperatures and their pinning strength is isotropic for different field directions [15]. A second example is superconducting tapes for power cables which require pinning sites that are most effective near liquid nitrogen temperatures in fairly low unidirectional magnetic fields. In this situation 1D columnar defects have been shown to yield high critical current densities at 77 K, although in this case *J_c_* is highly anisotropic and varies strongly with the magnetic field direction [14]. In the end, combining different types of pinning sites may be the best approach to attain the ultimate HTS tape performance. The combination of all these factors makes it very challenging to predict the magnitude of pinning forces and *J_c_* in advance and materials scientists must rely heavily on several complementary characterisation methods to understand the roles played by different pinning centres. Advancing our understanding of the pinning potential landscape and the behavior of flux lines within it will enable greater control of the maximum attainable *J_c_* and allow these tapes to get closer to their ultimate theoretical limit set by the pair-breaking current density [16].

A large number of academic research groups and industrial manufacturers are developing high-performance 2G-HTS tapes using different approaches [17]. Here, we investigate commercial high-*J_c_* GdBa_2_Cu_3_O_7−δ_ (GBCO)-based tapes fabricated by the manufacturer SuNAM using reactive co-evaporation [18,19]. These tapes have exceptional critical current densities well in excess of 1 MAcm^−2^ at 77 K under self-field conditions. In addition, *T_c_* lies in the range 92–95 K, well above liquid nitrogen temperatures. This is achieved by careful tuning of BaO, CuO_z_ and Gd_2_O_3_ second-phase inclusions produced during a deliberately Cu-rich deposition [20]. Applications for these tapes in motors, generators and magnet inserts require a high *J_c_* in high external fields, and to meet this need, the microstructure of Gd_2_O_3_ nanoparticles is optimised to significantly raise the in-field *J_c_*. However, if the density of pinning centres becomes too high, their performance degrades again and the precise tuning of the Gd to Cu-Ba ratio is required to attain optimal HTS tapes. Transmission electron microscopy (TEM) images of such tapes show uniformly dispersed, almost platelet-like Gd_2_O_3_ second-phase inclusions in the best performing tapes [18,19]. This contrasts with the more random and enlarged Gd_2_O_3_ nanoparticles found in worse performing in-field tapes. The establishment of the optimum growth conditions is very challenging, requiring the microscopic characterisation of the vortex pinning landscape and its interpretation in terms of the nanoscale pinning centres known to be present.

Transport and magnetisation measurements are two of the most common tools available to tape developers to characterise the ensemble-averaged pinning landscape at a macroscopic scale. In contrast, scanning probe microscopy techniques enable one to build a microscopic picture of the superconducting properties and represent ideal tools to study vortex behavior at discrete pinning centres [21]. Scanning Hall probe microscopy (SHPM) is a quantitative and non-invasive magnetic imaging technique that has frequently been used to study supercurrent transport [22], ac losses [23] and flux penetration in cuprate superconductors at the microscale [24]. In this paper, we use SHPM, supported by magnetisation and electronic transport measurements, to obtain a microscopic picture of the vortex pinning landscape at very low applied fields across a wide temperate range from 10 to 89 K. We couple this with scanning electron microscopy (SEM) imaging to attempt to develop a detailed understanding of the role second-phase inclusions play in pinning vortices and enhancing *J_c_*. These studies reveal dense and inhomogeneously distributed nanoscale pinning sites throughout the GBCO film, but with dramatically broadened vortex profiles that exhibit negligible temperature dependence down to 10 K. We attribute the exceptionally high critical current densities in these second-generation HTS tapes to the strong 1D pinning of individual vortex lines by multiple Gd_2_O_3_ nanoparticles. We tentatively suggest that the observed broadening of superconducting vortex fields across the entire studied temperature range arises from the substantial disruption of the supercurrent flow around the vortex cores due to the complex microstructure of the superconducting layer, greatly expanding their range. Such a scenario would clearly rely crucially on the shape and spatial distribution of pinning centres and provides important design rules for further enhancement of *J_c_* in the future.

## 2. Materials and Methods

Magnetic imaging was performed by scanning Hall probe microscopy (SHPM) (Nanomagnetics Instruments, Ankara, Turkey) in a commercial Oxford Instruments (OI) cryostat (Oxford Instruments, Oxford, UK). A nanoscale Hall probe was patterned in a GaAs/AlGaAs heterostructure 2D electron gas ~5 μm from the gold-coated corner of a deep mesa etch acting as a makeshift scanning tunneling microscope (STM) tip. The STM tip was used to approach the sensor to the sample surface and track it once the two were in close proximity. The ultimate spatial resolution of the Hall sensor was defined by the electronic wire width of the two intersecting Hall cross leads. A Hall probe with an electronic width of 500 nm was used for the images obtained in Figures 4 and 5, while a Hall probe with an electronic width of 800 nm was used for the images obtained in Figure 6. For Figure 7 the 500 nm electronic width Hall probe was returned to. To ensure that the Hall probe was in the closest possible proximity to the superconducting layer, a 2-μm-thick protective Ag layer was removed from a 6 mm length of 4-mm-wide 2G-HTS tape by wet chemical etching with ammonium peroxide. The tape was subsequently recoated with a Cr/Au (5 nm/40 nm) film to ensure good electrical contact between the sample and the STM tip and epoxied onto a silicon substrate. The Hall probe was mounted at an angle of approximately 1° with respect to the coated tape such that the STM tip is always the closest point to the tape surface [25]. A piezoelectric stick-slip driver and scanner tube facilitated the automated approach of the Hall probe towards the sample until a threshold current of 0.2 nA was reached with the sample usually biased at +0.2 V with respect to the grounded STM tip. The Hall probe was then lifted approximately 50 nm above the sample surface for rapid “flying-mode” imaging to produce a 2D map of the magnetic induction. Several images were taken and averaged frame-by-frame to suppress the low-frequency noise from the Hall probe. Different external magnetic fields could be applied from either a normal Cu wire coil wound around the cryostat or a superconducting magnet inside it. The scan range of the piezoelectric tube is strongly temperature-dependent and varies from 8.5 µm × 8.5 µm to 22.4 µm × 22.4 µm between 10 and 88 K. Local magnetometry was performed by parking the Hall sensor ~1 µm above a desired location on the superconducting sample and measuring the magnetic induction as an external field was swept around a hysteresis loop. At very large sample biases, the high electric field associated with tall topographic features on the sample also modulates the response of the Hall sensor, and the structural information obtained from such “gating” images can then be directly correlated with magnetic information from the same location. In practice, these images were generated by subtracting images captured at sample bias voltages of +2 V and −2 V, resulting in the removal of magnetic features and leaving only the topographic information of interest.

Electrical transport measurements were performed in a standard 4-point measurement configuration at temperatures down to 90 K. Prior to Ag removal, two pairs of leads were soldered with a fixed spacing along a 16 mm strip of 2G-HTS tape. These were subsequently contacted with the pads of a 20-pin ceramic package which was then attached to the end of a temperature-controlled sample holder designed to fit inside the OI cryostat. A constant current of 10 mA was passed through the strip while the voltage was measured using a Keithley 182 nanovoltmeter as the temperature was swept at 0.1 K/min using an OI ITC 503 temperature controller.

Field effect scanning electron microscopy (FE-SEM) at 5 and 1 kV and energy-dispersive X-ray analysis (EDX) at 10 kV were performed in a Joel JSM-6301F FE-SEM (JOEL, Tokyo, Japan). Focused ion beam (FIB) milling for cross-sectional imaging of a tape sample was performed in an FEI Helios NanoLab 600 (FEI Hillsboro, OR, USA), with electron images captured at an acceleration voltage of 5 kV.

All data captured within this research project are openly available from the University of Bath Research Data Archive [26].

## 3. Results and Discussion

### 3.1. Characterisation of the HTS Tapes

**Figure 1:**
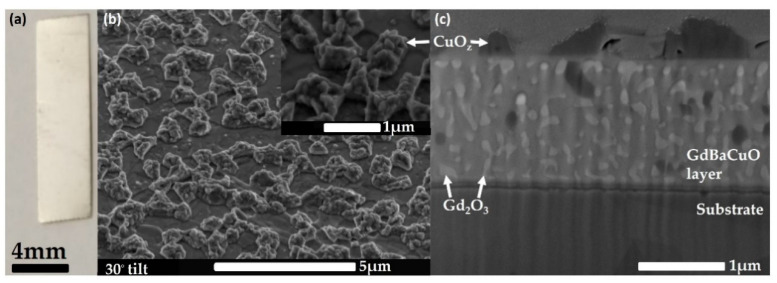
(**a**) Optical image of a strip of GBCO HTS tape covered with a 2-μm-thick Ag stabiliser layer; (**b**) An SEM image of the surface of the tape after stabiliser removal at a 30 degree tilt angle. The inset shows a magnified SEM image of a region from the larger area of (**b**), showing the presence of CuO_z_ growths on the upper layers of the superconducting film; (**c**) A cross-sectional electron micrograph of the GBCO layer showing the Gd_2_O_3_ nanoparticles (light) and CuO_z_ inclusions (dark). Above the GBCO layer, CuO_z_ outgrowths are visible embedded in a platinum coating deposited to protect the surface during FIB milling. The bottom layer is the Hastelloy tape substrate.

An example of an as-received piece of tape covered by a 2-μm-thick Ag stabiliser layer is shown in Figure 1a. The width of the HTS tape strip is 4 mm, while the thickness of the GBCO film below the stabiliser layer is approximately 1.5 µm. Once the stabiliser layer was etched away, the dark, reflective and seemingly flat superconducting layer was exposed. Upon closer inspection of the surface of this layer with SEM and EDX (cf. Figure S1), we find regions of sub-micrometre scale networks of non-superconducting CuO_z out_growths with the lightly terraced yet much flatter superconducting GBCO layer in between (cf. Figure 1b). After FIB milling through the superconducting layer, buffer layers and a portion of the Hastelloy substrate, we are able to image a cross-section of the GBCO layer. This is shown in Figure 1c, where light-coloured Gd_2_O_3_ nanoparticles are clearly resolved throughout the active GBCO layer of greatly varying sizes and shapes. More than half of these nanoparticles appear to be approximately circular isolated particles with typical radii in the range ~50–100 nm. However, larger Gd_2_O_3_ nanoparticles are also present that appear to join up, forming a more connected network of normal material throughout the superconducting layer. There are also a few dark inclusions within the superconducting layer. Since these have the same contrast as the CuO_z_ outgrowths at the surface, we assume that they have the same composition and represent a second source of normal pinning sites for vortices.

**Figure 2:**
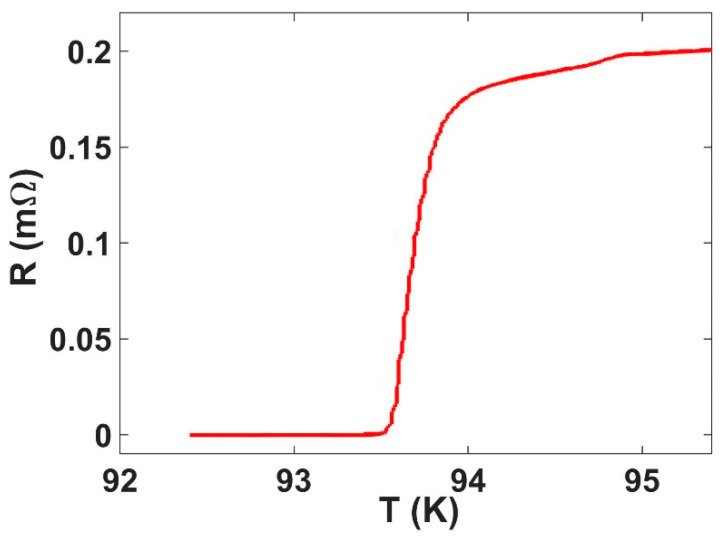
Four-terminal resistance measured as a function of temperature showing a mid-point resistive transition at 93.4 ± 0.2 K.

Temperature-dependent electronic transport measurements on a piece of tape with similar dimensions to the one in Figure 1a revealed a high mid-point resistive *T_c_* of 93.4 ± 0.2 K, as shown in Figure 2. This falls within the range of previous measurements on similar types of tape [18,19]. The kink feature observed just below 95 K is attributed to the loss of residual superconductivity in regions of the GBCO film with a slightly higher *T_c_* than average [18]. While these regions do not constitute a continuous percolating path through the sample, they can lead to a highly inhomogenous current flow, even when the bulk of the film is already in the normal state. This in turn leads to signatures in the temperature-dependent resistance of the tape that coincide with the loss of superconductivity in these very high T_c_ regions.

The *J_c_* for our tapes was estimated from local measurements of the magnetisation using the parked SHPM Hall sensor, after fitting to a “two-dimensional” critical state model developed by Brandt and Indenbom [27]. This describes the magnetic field penetration profile, *B_z_*, in a zero field-cooled infinitely long type-II superconducting strip of width 2*a*, and thickness *d*, under a constant perpendicular externally applied magnetic field. Within the assumptions of the model (*d* << 2*a*), the critical current density can then be extracted as the sole unknown parameter. At an applied field *H_a_*, the magnetic field profile is described by

(1) $$B_{z}\left(y\right)=\{\begin{array}{cc} 0 & \left|y\right|<b\\ B_{c}\;tanh^{-1}\frac{\sqrt{y^{2}+b^{2}}}{c\left|y\right|} & b<\left|y\right|<a\\ B_{c}\;tanh^{-1}\frac{c\left|y\right|}{\sqrt{y^{2}+b^{2}}} & \left|y\right|>a \end{array},\;$$

where *B_c_* = *µ*_0_*J_c_d/π*, *c = tanh(µ*_0_*H_a_/B_c_)* and *b = a/cosh(µ*_0_*H_a_/B_c_)* is the boundary of the central flux-free region. As the sweep direction of the applied field reverses again from *H_max_*, *B_z_*(*y*) can be calculated from [27]

(2) $$B_{z}^{↓}\left(y,H_{a},J_{c}\right)=B_{z}\left(y,H_{max},J_{c}\right)-B_{z}\left(y,H_{max}-H_{a},2J_{c}\right).$$

**Figure 3:**
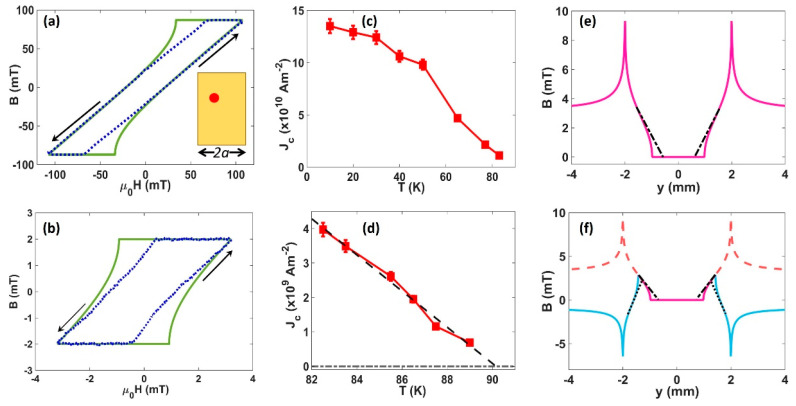
(**a**) B-H loop at 65 K (dotted line) and a fit to the 2D critical state model (solid line). Arrows indicate the direction of the field sweep. The inset shows a sketch of the tape sample showing the position where local magnetometry measurements were performed (red dot); (**b**) B-H loop at 84 K (dotted line) and a fit to the 2D critical state model (solid line). Arrows indicate the direction of the field sweep; (**c**) temperature-dependent critical current density estimated from fitting magnetisation loops swept out to ±107 mT; (**d**) temperature-dependent critical current density estimated from magnetisation loops swept out to ±3.2 mT. These data were used to estimate the critical temperature from a linear extrapolation (dashed line) to *J_c_* = 0. (**e**) Magnetic field profile predicted by the 2D critical state model when the external field is increased from zero to *μ*_0_.*H_max_* = 3.2 mT (*J_c_ =* 5 × 10^9^ A/m^2^). The superimposed dash-dot line illustrates the actual behavior of the penetration front exhibited by our sample; (**f**) model magnetic field profile after the applied field in (**e**) has been reduced again to *μ*_0_.*H_max_* = −0.94 mT (solid lines). The dashed red line shows the original penetration profile at the maximum applied field. The superimposed dotted line illustrates the actual behavior of the repenetration front exhibited by our sample.

The Hall probe was positioned approximately *y* = 0.74 mm from the centre of the strip, and magnetisation loops were captured using external field excursions *H_max_* = ±107 mT from 10 up to 83 K and *H_max_* = ±3.2 mT from 81 up to 87.5 K. The two different sweep ranges were used to ensure sufficient resolution in the hysteretic B-H loops across the full temperature range of interest. Example B-H loops and fits to the model are shown in Figure 3a,b. The resulting estimations of *J_c_* as a function of temperature are plotted in Figure 3c for higher field sweeps, and in Figure 3d for the lower field sweeps. The critical temperature can also be estimated from a linear extrapolation of *J_c_* at high temperatures, yielding a value of 90.2 ± 0.2 K, as indicated in Figure 3d. The differences between the resistive *T_c_* and magnetic estimations of *T_c_* probably reflect small inhomogeneities in the film since the resistive measurement detects the strongest percolation path through the entire sample, while the magnetisation data are only collected from the “local” area near the Hall sensor. The estimated critical current density at 77 K is within the range of previously estimated values obtained from transport measurements at the same temperature, with any difference likely to be due to the different in-field and self-field measurement conditions used [18,19,28]. Estimated *J_c_* values for the smaller field range near *T_c_* are systematically smaller than those for the larger sweep range, reflecting the much lower sweep rates (approximately 30x lower) for these loops, allowing much more time for the critical state to relax due, for example, to flux creep processes. These nevertheless represent exceptionally high critical current densities in comparison to other 2G-HTS tapes, reflecting the presence of very strong vortex pinning forces. A comparison between the data and the model fits in Figure 3a,b reveals significant discrepancies, particularly at the points where the reverse flux repenetrates the sample. In practice, the reverse flux penetrates much sooner than expected from the model and does not exhibit the very abrupt predicted onset. While our samples adequately satisfy the model assumption that *d* << 2*a*, the strips we measure are only of finite length with an aspect ratio of ~3:2 and the critical current is microscopically inhomogeneous on a scale of the distribution of artificial pinning centres. However, neither of these shortcomings seem able to explain the discrepancies with the fits which are more likely to be related to the relaxation of critical state profiles by flux creep. To illustrate this point, in Figure 3e,f, we plot the magnetic induction profiles predicted by the model after increasing the applied field from zero to the maximum excursion, *μ*_0_.*H_max_* = 3.2 mT, and, after subsequent field reversal, to −0.94 mT, respectively. Our measured data are quite closely represented by the dash-dot and dotted lines that have been superimposed to represent the smearing of the penetrating and repenetrating flux fronts due to flux creep. In fact, the profile presented by these lines much more closely mirrors the classical 3D Bean critical state behavior whereby d*B_z_*/d*y* is a constant for a given sign of the critical current density [27]. A similar behavior has been observed in local magnetisation data measured on other types of 2G-HTS tapes [24], though not to the same extent as that seen here, indicating a significantly faster relaxation rate in our samples. This in turn reflects the type, structure and topology of the specific pinning centres employed which lead to distinctly different creep barriers for different configurations.

### 3.2. Vortex Pinning Landscape in Low Fields

**Figure 4:**
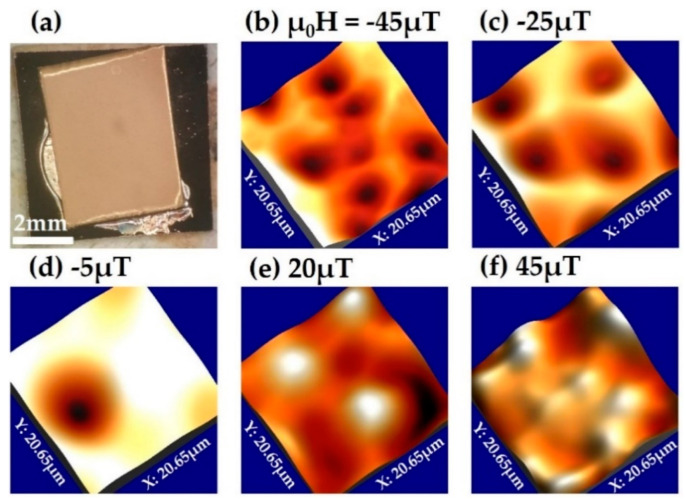
(**a**) Optical microscope image of the section of GBCO 2G-HTS tape imaged after the Ag stabiliser layer has been removed, and the sample then recoated with Cr/Au. The width, 2*a* = 4 mm, of the tape is given by its shortest length of 4 mm; (**b**–**f**) three-dimensional renderings of SHPM vortex images captured on the GBCO tape after field cooling to 77 K from above *T_c_* in perpendicular applied fields between −45 and 45 µT. The scan size is 20.7 × 20.7 µm. Vertical scales span 40 µT (μ_0_H = −45 µT), 23 µT (−25 µT), 23 µT (−5 µT), 16 µT (20 µT) and 40 µT (45 µT).

To further our understanding of the relationship between vortex pinning and the film microstructure, we systematically captured a series of SHPM images of a sample of the 2G-HTS tape after field cooling in small (<1 mT) perpendicular magnetic fields. Figure 4 shows a series of 3D renderings of vortex images captured at 77 K after field-cooling from above *T_c_* in several different fields [29]. We note that the applied fields stated are nominal values for the coil currents used, and in practice, the sample also sees contributions from the earth’s field, nearby ferrous materials (e.g., the Hastelloy substrate of the tape [30]) and flux trapped in the windings of the superconducting magnet. As expected for a strong pinning film, vortex patterns are highly disordered and the number of first nearest neighbours frequently deviates from that expected for a perfect Abrikosov vortex lattice (i.e., six). In addition, specific sites are repeatedly occupied by a vortex after different field cooling cycles, suggesting that a few particularly strong pinning centres are present.

**Figure 5:**
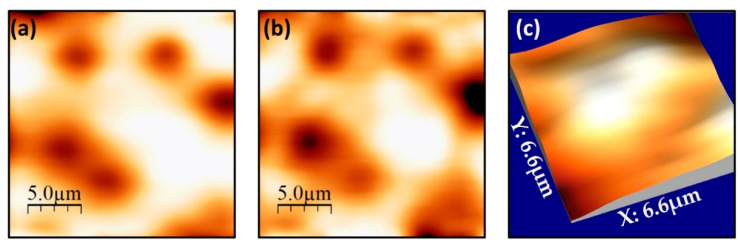
(**a**,**b**) SHPM images of vortices in a GBCO 2G-HTS tape after field cooling to 77 K from above *T_c_* in the same applied perpendicular field of μ_0_H ≈ −40 µT. The vertical scales span 40 µT and the scan size is 20.7 × 20.7 µm; (**c**) An expanded view of two very close white vortices in the centre of the sample after field cooling to 77 K in μ_0_H ≈ 95 µT, which were used to obtain a lower bound estimate of the pinning. The vertical scale spans 26 µT.

Figure 5a,b examine the reproducibility of vortex patterns after repeatedly field cooling the sample in the same applied field. As can be seen, the vortex pattern is highly reproducible from one cooldown cycle to the next, indicating that a small number of very strong pinning sites with a wide capture radius are dominating these frozen structures. This is in stark contrast to weakly pinning materials where such patterns tend to change dramatically between different thermal cycles due to statistical fluctuations in the freezing dynamics during the cooldown.

A lower bound for the pinning force experienced by vortices was estimated from two very close vortices in the image shown in Figure 5c. Assuming that both vortices are pinned with a force that matches or exceeds their mutual repulsion, we compare this with an expression for the force between two vortices in a bulk superconductor when the vortex–vortex separation, *r*, is much greater than the London penetration depth, *λ_L_*. This is described by [31]

(3) $$f_{p}^{v\to v}\approx \frac{\Phi_{0}^{2}}{2\pi {µ}_{0}\lambda_{L}^{2}}\sqrt{\frac{\pi }{2\lambda_{s}r}}e^{\frac{-r}{\lambda_{L}}},$$

where *Φ*_0_ is the superconducting flux quantum. We estimate that two vortices in Figure 5c are ~1.6 µm apart, yielding a lower bound on the pinning force of *f_p_*~0.035 µNm^−1^. In practice of course, the spatial resolution of our imaging technique (~0.5 μm) severely limits our ability to resolve two vortices at very close spacing and this number is inevitably a huge underestimate of typical pinning forces at this temperature. Moreover, it is known that the vortex structure freezes at a typical “irreversibility temperature” just below *T_c_*, where pinning forces become just strong enough to quench the thermally activated vortex motion. Hence, these vortex patterns are characteristic of even weaker pinning forces that apply much closer to *T_c_*. A much more rigorous estimate of the average pinning force can be obtained from the relationship *f_p_ ≈ J_c_Φ*_0_. Using the estimate of *J_c_* at 77 K from Figure 3d, we use this expression to calculate that pinning forces are approximately *f_p_*~44 µNm^−1^, close to other high-*J_c_* cuprate materials and 2G-HTS tapes at similar fields [18,19,32,33]. As expected, this is several orders of magnitude larger than the force estimated from the separation of discrete vortices in SHPM images.

**Figure 6:**
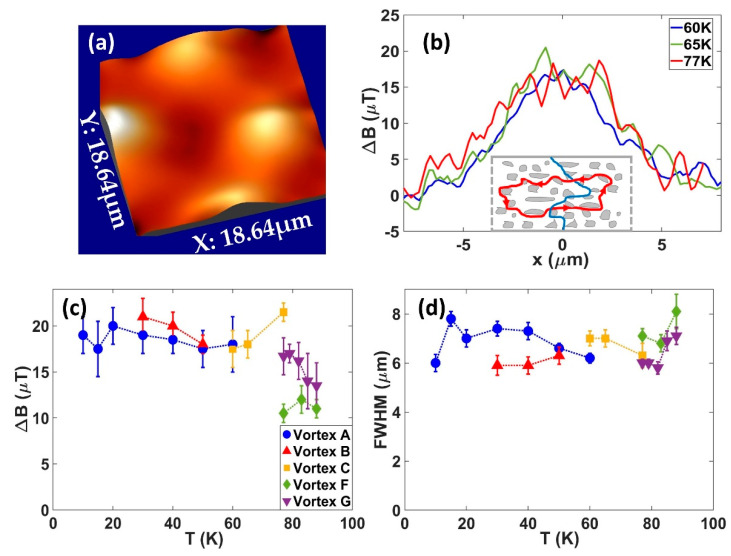
(**a**) Three-dimensional rendering of an SHPM image of the vortex pattern after field cooling to 65 K μ_0_H = 25 µT. The vertical scale spans 19 µT and the scan size is 18.6 × 18.6 µm; (**b**) Typical vortex profile linescans across the centre of vortex C at three different temperatures. The inset shows a sketch of a pinned vortex core (blue) surrounded by supercurrents (red) with strongly distorted and more widely distributed trajectories due to the presence of the high density of Gd_2_O_3_ pinning centres (grey); (**c**) Peak heights of vortex profiles as a function of temperature for several different pinned vortices; (**d**) FWHM of vortex profiles as a function of temperature for the same vortices shown in (**c**).

The quantitative nature of SHPM imaging also allows us to conduct a careful analysis of temperature-dependent vortex profiles which have been characterised in images of the type shown in Figure 6a. Figure 6b illustrates linescans captured across the centre of one of these vortices at three different temperatures. Data for several different pinned vortices are summarised in Figure 6c,d in terms of the peak vortex heights and the full-width-half-maxima (FWHM), respectively. It is really striking that neither figure of merit shows any significant temperature dependence between 10 and 85 K, with only a weak additional broadening of the vortices closer to *T_c_*. Attempts to fit these profiles to the variational model predictions for type-II superconductors due to Clem fail completely, even if one assumes an unphysically large variational coherence length that breaks the assumption *λ_L_* >> ξ_v_ [34,35]. In the limit that the measured FWHM of the vortex is governed by the diameter of the non-superconducting centre it is pinned on, these very large temperature-independent sizes might be possible. However, Gd_2_O_3_ nanoscale inclusions that are believed to dominate vortex pinning only have sizes of a few hundred nanometres, as seen in Figure 1c and in [18,19], orders of magnitude smaller than the vortex diameters we measure. To understand this observation, we first analyse the nature of the vortex pinning in our tapes in more detail. By comparing the density of pinning sites with the 1D pinning density threshold, *n*_1*D*_*~16π*^2^*λ*^2^*fp/Φ*^2^_0_*ξ*^3^, derived by Blatter et al. [36], we deduce that the images in Figure 4 and Figure 5 were captured in the 1D strong pinning regime. In this limit, individual vortices are pinned at several different pinning sites along their length and can meander somewhat between them. However, the additional vortex line energy required will limit the overall degree of lateral wandering to being less than the thickness of the superconducting layer (1.5 µm) and cannot explain the degree of broadening observed in our images. Moreover, since we are dealing with strong pinning sites and find almost no temperature dependence of the vortex profiles below 80 K, we can rule out dynamic broadening due to thermal hopping of vortex segments between different pinning sites. Hence, we tentatively suggest that the broadening arises from substantial disruption of the supercurrents flowing around the vortex core due to the complex microstructure of the superconducting layer and the high density of second-phase pinning sites it contains. This is reflected in the substantial extension of the range of the vortex magnetic fields, as seen in the magnetic images. The inset of Figure 6b qualitatively illustrates this scenario.

### 3.3. Correlating Pinning Sites

**Figure 7:**
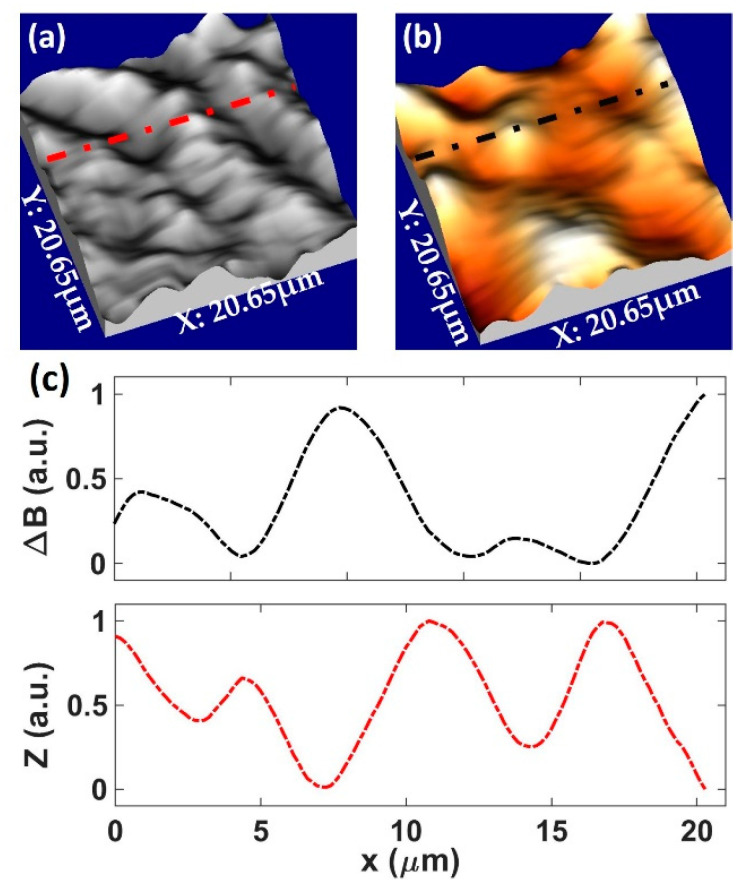
(**a**) Three-dimensional rendering of a topographic “gating” image captured at 77 K; (**b**) three-dimensional rendering of vortices at the same location as (**a**). Images were captured after field cooling to 77 K in μ_0_H = 25 µT. The vertical scale for (**b**) spans 15 µT. Scan sizes for all images are 20.7 × 20.7 µm. (**c**) Shows linescans along the dashed-dotted lines superimposed on images (**a**) and (**b**).

One of the challenges when it comes to developing 2G-HTS tapes is to understand which pinning objects in the superconducting film are dominating any increase in the critical current. SEM images of these SuNAM tapes reveal both a high density of quite uniformly dispersed multilayer Gd_2_O_3_ inclusions with a wide range of orientations throughout the superconducting layer and much larger CuO_z_ outgrowths on the top surface, as shown in Figure 1b [18,19]. Since the latter tend to have much larger lateral dimensions and are also non-superconducting, it is important to rule out that these are not also contributing significantly to the pinning of superconducting vortices. Due to their very large size compared to the superconducting coherence length, we expect these to behave as 3D pinning sites at medium to high magnetic fields. As these features are found at the tape surface, they represent the main contribution to the surface topography and can be mapped using the “gating” imaging approach described in Section 2. In this way, we can search for correlations between the location of the CuO_z_ outgrowths and the locations of strongly pinned vortices. Figure 7a shows examples of topographic maps that were captured just after the magnetic maps of Figure 7b (measured with a normal sample bias of 0.2 V). Comparing the pairs of images, we are unable to find any significant correlation between the largest topographical features and the characteristic vortex pinning locations. As clearly seen in the linescans plotted in Figure 7c, the vortex centres do not appear to bear any relationship with the measured topographic peaks (or troughs), something that has been confirmed by generating a numerical cross-correlation of the image pairs. Hence, we consider it very unlikely that these non-superconducting CuO_z_ components make a significant contribution to the vortex pinning landscape, and they are hence inactive in the measurement regimes we explore. Instead, everything points to the fact that the Gd_2_O_3_ nanoparticles distributed throughout the superconducting layer play the dominant role in maximising *J_c_*.

## 4. Conclusions

Our results yield important new insights into the nature of vortex pinning in commercial GBCO high-temperature superconducting tapes, providing a new understanding that can be built on to further increase *J_c_* in these tapes. Scanning Hall probe microscopy images reveal highly disordered vortex patterns, which have not previously been observed in GBCO HTS tapes, reflecting a pinning landscape dominated by dense nanoscale pinning centres. These pinning centres are most likely non-superconducting Gd_2_O_3_ second-phase inclusions acting as strong 1D pinning sites at low magnetic fields. Furthermore, local magnetisation loops have, for the first time, shown surprising deviations from the predictions of the widely accepted 2D Bean critical state model for infinite superconducting strips, suggesting that penetration and repenetrating flux profiles relax rather rapidly due to flux creep. Indeed, we infer that our measured profiles are closer to the predictions of the bulk 3D Bean critical state model with approximately constant values of d*B_z_*/d*y* for a given sign of the critical current density. Unexpectedly, and not previously observed, we measured extremely broad vortex profiles with FWHM typically of 6 μm. Moreover, neither the vortex peak fields nor the FWHM show significant temperature dependence in the range 10–85 K. These facts are very difficult to reconcile with the sizes of the dominant Gd_2_O_3_ pinning centres which are typically a few hundred nanometres in diameter. This broadening cannot be explained in terms of the static or dynamic (i.e., hopping) meandering of the vortex core between pinning sites and we tentatively attribute it to the substantial disruption of the supercurrent flow around a vortex core due to the dense distribution of nanoscale pinning sites, greatly extending the range of the associated magnetic fields. This introduces an important new aspect to consider for the future development of HTS tapes using second-phase pinning to increase *J_c_*. Our work also reinforces the role magnetic imaging has as a powerful complementary tool for developing and optimising high-temperature superconducting tapes and opens up opportunities for further studies of flux pinning in GBCO tapes under self-field conditions [37]. In addition, ongoing improvements in the spatial resolution of magnetic imaging techniques [38,39] will allow investigations to be performed with greater precision in order to resolve pinning sites in superconducting tapes across a much wider range of temperatures, external fields and applied currents to determine how effective these pinning sites are in different regimes. This investigative approach would become even more powerful when combined with simultaneous imaging of cross-sections of the superconducting layer to more accurately correlate the locations of vortices and pinning sites.

## Data Availability

All data captured in the course of this research work are openly available from the University of Bath Research Data Archive at https://doi.org/10.15125/BATH-00945, accessed on 21 April 2021.

## Supplementary Materials

The following are available online at https://www.mdpi.com/article/10.3390/nano11051082/s1, Figure S1: EDX analysis of the GBCO layer surface.
