# Supplementary material for: Imaging of Strong Nanoscale Vortex Pinning in GdBaCuO High-Temperature Superconducting Tapes
Source: Nanomaterials (Basel). 2021 Apr 22;11(5):1082. doi: 10.3390/nano11051082 (PMC8145501; doi:10.3390/nano11051082)
Supplement: Supplementary file 1 [file nanomaterials-11-01082-s001.zip › nanomaterials-1167648-supplementary.pdf]

nanomaterials

Supporting Information

Imaging of Strong Nanoscale Vortex Pinning in GdBaCuO High-Temperature Superconducting Tapes

David Collomb [1,*], Min Zhang [2], Weijia Yuan [2] and Simon J. Bending [1]

[1] University of Bath, Department of Physics, Claverton Down, Bath BA2 7AY, UK; pyssb@bath.ac.uk
[2] Applied Superconductivity Laboratory, Department of Electronics and Electrical Engineering, University of Strathclyde, Glasgow G1 1XQ, UK; min.zhang@strath.ac.uk (M.Z.); weijia.yuan@strath.ac.uk (W.Y.)
* Correspondence: dc805@bath.ac.uk

1. EDX Analysis of the GdBaCuO Layer Surface

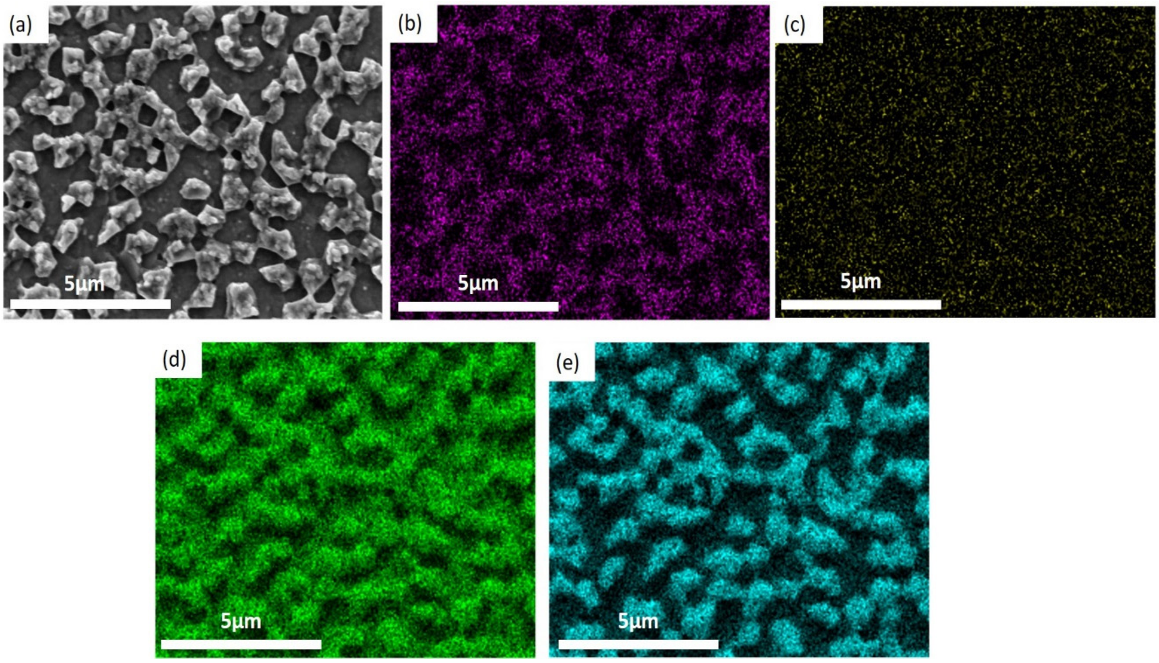

Figure 1. (**a**) Reference SEM image of the area where EDX was been performed.; (b) EDX map of Barium distribution of intensity.; (c) EDX map of Gadolinium distribution intensity.; (d) EDX map of Oxygen distribution of intensity.; (e) EDX map of Copper distribution of intensity.
